# Decreased EAAT2 protein expression in the essential tremor cerebellar cortex

**DOI:** 10.1186/s40478-014-0157-z

**Published:** 2014-11-13

**Authors:** Michelle Lee, Melody M Cheng, Chi-Ying Lin, Elan D Louis, Phyllis L Faust, Sheng-Han Kuo

**Affiliations:** GH Sergievsky Center, College of Physicians and Surgeons, Columbia University, New York, NY USA; Department of Neurology, College of Physicians and Surgeons, Columbia University, New York, NY USA; Taub Institute for Research on Alzheimer’s Disease and the Aging Brain, College of Physicians and Surgeons, Columbia University, New York, NY USA; Department of Epidemiology, Mailman School of Public Health, Columbia University, New York, NY USA; Department of Pathology and Cell Biology, Columbia University Medical Center and the New York Presbyterian Hospital, New York, NY USA

**Keywords:** Essential tremor, EAAT1, EAAT2, Excitotoxicitiy, Purkinje cell, Neurodegenerative

## Abstract

Genetic polymorphisms in *Solute carrier family 1 (glial high affinity glutamate* transporter), member 2 (SLC1A2) have been linked with essential tremor. *SLC1A2* encodes excitatory amino acid transporter type 2 (EAAT2), which clears glutamate from the synaptic cleft. One postulated mechanism for essential tremor is the over-excitation of glutamatergic olivo-cerebellar climbing fibers, leading to excitotoxic death of Purkinje cells. Other glutamatergic excitatory signals are transmitted to Purkinje cells via parallel fibers of cerebellar granule neurons. Therefore, the expression level of glutamate transporters could be important in essential tremor pathogenesis. Using Western blotting, we compared the expression levels of the two main glutamate transporters in the cerebellar cortex, EAAT1 and EAAT2, in postmortem tissue from 16 essential tremor cases and 13 age-matched controls. We also studied the localization of EAAT1 and EAAT2 using immunohistochemistry in 10 essential tremor cases and 12 controls. EAAT1 protein levels were similar in cases and controls (1.12 ± 0.83 vs. 1.01 ± 0.69, p =0.71) whereas EAAT2 protein levels in essential tremor cases were only 1/3 of that in controls (0.35 ± 0.23 vs. 1.00 ± 0.62, p < 0.01). Interestingly, EAAT2, but not EAAT1, was expressed in astrocytic processes surrounding the Purkinje cell axon initial segment, a region of previously observed pathological changes in essential tremor. Our main finding, a significant reduction in cerebellar cortical EAAT2 protein levels in essential tremor, suggests that Purkinje cells in essential tremor might be more vulnerable to excitotoxic damage than those of controls.

**Electronic supplementary material:**

The online version of this article (doi:10.1186/s40478-014-0157-z) contains supplementary material, which is available to authorized users.

## Introduction

Essential tremor (ET) is a prevalent neurological disease marked by a persistent 4-12 Hz action tremor in the arms [1]. ET has a strong genetic component, as ET patients often have a family history of tremor, and twins with ET are highly concordant for disease status [2]. Recently, polymorphisms in the *solute carrier family 1 (glial high affinity glutamate transporter), member 2* (*SLC1A2*) gene have emerged as a potential genetic risk factor for ET in a genome-wide association study in Europe [3]. While this association was subsequently confirmed in two Asian cohorts [4,5], another study did not show an association [6], and a meta-analysis revealed conflicting results [7]. Given this evolving picture, the *SLC1A2* gene remains of considerable interest. The *SLC1A2* gene encodes excitatory amino acid transporter 2 (EAAT2), which is a protein that is critical for maintaining glutamate levels in the synaptic cleft in the adult brain [8].

Glutamatergic synapses are the major excitatory synapses in the brain, and brain glutamate levels are exquisitely controlled [8]. Excessive extracellular glutamate, due to over-excitation of neurons or failure of glutamate recycling, can lead to mitochondrial dysfunction and subsequent neuronal death. This mechanism of “excitotocity” has been implicated to be of potential patho-mechanistic importance in epilepsy, stroke, and amyotrophic lateral sclerosis [9-11]. Extracellular glutamate levels are mainly regulated by a family of glutamate transporters, the excitatory amino-acid transporters (EAATs), which in the central nervous system comprise five subtypes (EAAT1 - 5) [12]. Both EAAT1 and EAAT2 are expressed predominantly in astrocytes, the major cell type responsible for glutamate uptake [13]. EAAT1 is expressed during development and also in adulthood, whereas EAAT2 is the main glutamate transporter in adult brain, responsible for over 90% of total glutamate uptake [8,12]. EAAT3, EAAT4, and EAAT5 are localized primarily in neurons, and may play a role in neuronal excitability [8].

Clinical and neuroimaging evidence has pointed to the importance of the cerebellum in the pathogenesis of ET [14]. Postmortem studies have revealed a broad range of structural changes in the cerebellum in ET. These include Purkinje cell (PC) loss in some studies [15,16], but not in the others [17,18]. One hypothesized mechanism for ET is that it is a disorder of over-excitation of glutamatergic olivo-cerebellar climbing fibers, which results in excitotoxic damage to PCs. Alterations in EAAT levels in the cerebellar cortex could result in a breakdown in normal extracellular glutamate homeostasis, enhancing vulnerability to excitotoxic damage. Since EAAT1 and EAAT2 are the two major glutamate transporters in the cerebellar cortex, we systematically investigated the expression level and immunohistochemical cellular localization of these two proteins in the postmortem cerebellum of ET cases vs. controls.

## Materials and methods

### Brain repository and study subjects

The study was conducted at the Essential Tremor Centralized Brain Repository (ETCBR), New York Brain Bank (NYBB), Columbia University, New York. Post-mortem tissue was obtained from ET cases and age-matched non-diseased controls, as previously described [19].

The clinical diagnosis of ET was initially assigned by treating neurologists, and then confirmed by an ETCBR study neurologist using medical records, study questionnaires, a detailed videotaped neurological assessment, and ETCBR diagnostic criteria [20]. The study questionnaires included data on family history of tremor and medications (Additional file 1: Table S1). The neurologist (EDL) reviewed all videotaped neurological examinations and rated the severity of postural and kinetic (pouring, drinking, using spoon, drawing spirals, finger-nose-finger) arm tremors (ratings =0 - 3), resulting in a total tremor score (range =0 to 36 [maximum]) [21]. The presence of head or voice tremor on examination was noted in each patient. None of the ET cases or controls had a history of traumatic brain injury or heavy ethanol use, as previously defined [22]. Non-diseased control brains were obtained from the NYBB and were from individuals followed at the Alzheimer disease (AD) Research Center or the Washington Heights Inwood Columbia Aging Project at Columbia University. They were followed prospectively with serial neurological examinations, and were clinically free of AD, ET, Parkinson’s disease, Lewy body dementia, or progressive supranuclear palsy.

We selected available age-matched, non- diseased control brains from NYBB based on age at death. Therefore, cerebellar cortical tissue was available for immunohistochemistry on 10 ET cases and 12 controls with a similar age distribution, and for Western blot analyses on 16 ET cases and 13 controls with a similar age distribution. There was available banked tissue for both Western blot and immunohistochemistry experiments in 8 ET brains and 9 control brains.

As previously described, all ET and control brains had a complete neuropathological assessment at the NYBB [19]. Brains had standardized measurements of brain weight (grams), postmortem interval (PMI, hours between death and placement of brain in a cold room or upon ice). We excluded ET cases with Lewy body pathology (α-synuclein staining).

A standard 3 × 20 × 25 mm parasagittal neocerebellar block was obtained from a 0.3-cm-thick parasagittal slice located 1 cm from the cerebellar midline. Paraffin sections (7 um thick) were stained with Luxol fast blue hematoxylin and eosin (LH&E) or Bielschowsky silver methods as described previously [15]. Axonal torpedoes were quantified in the entire LH&E-stained section.

### Cerebellar immunohistochemistry

Seven-μm-thick paraffin-embedded cerebellar sections were incubated with polyclonal goat anti-EAAT1 antibody (Santa Cruz, sc-7758, 1:1000) or guinea pig anti-EAAT2 antibody (Millipore, ab1783, 1:200) at 4°C for 24 hours after antigen retrieval in Trilogy (Cell Marque) in a vegetable steamer for 60 minutes, 100°C. The sections were incubated with goat anti-rabbit (Fisher Scientific, 1:200) or anti-guinea pig IgG biotin-conjugated antibody (Millipore, 1:200), respectively, followed by 3,3′-diaminobenzidine (DAB) precipitation. We tested the immunohistochemical specificity of both EAAT1 antibody and EAAT2 antibody using EAAT1 or EAAT2 peptide block (EAAT1 peptide from Santa Cruz, sc-7758P, and EAAT2 peptide from Tocris Bioscience, 2128). For peptide block experiments, peptides and antibodies at 10:1 molar ratio or antibodies alone were incubated at 4°C for 24 hours and were then used as the primary antibody for immunohistochemistry. Microscopic images of immunohistochemically stained sections were obtained by bright field microscopy (Zeiss). EAAT2 immunopositive staining intensity in the cerebellar cortex was quantified in DAB-light microscopic cerebellar sections, and all EAAT2 immunohistochemistry was performed in the same batch with identical time of DAB precipitation. Automatic slide scanning was performed in each case and the intensity of EAAT2 immunoreactivity was measured with Leica Beiosystems Tissue IA Software, version 2.0. The intensity of EAAT2 immunoreactivity in the cerebellar cortex was normalized to that in the cerebellar white matter. All morphological measurements were performed by a rater (ML) blinded to the diagnosis, with a piece of colored tape coded with an English alphabet character covering the identification number on each slide, and random ordering of ET case and control specimens within the alphabetic blinded code.

For dual immunofluorescence studies, we labeled paraffin-embedded sections with anti-EAAT1 antibodies or anti-EAAT2 antibodies and mouse anti-glutamine synthetase (BD Transduction, 610518, 1:300), mouse anti-glial fibrillary acidic protein (GFAP) (Sigma G3893, 1:100), or rabbit anti-Lingo-1 antibody (Millipore, 07-678, 1:100). The secondary antibodies were goat anti-rabbit antibodies conjugated with Alexa fluorophore 488 or goat anti-mouse antibodies conjugated with Alexa fluorophore 594 (All Invitrogen, 1:100). Microscopic images were obtained by confocal microscopy (Leica).

### Western blot analysis

Frozen cerebellar cortex in standardized vials was solubilized in RIPA buffer (Sigma) with proteinase inhibitors (Roche Diagnostics) and phosphatase inhibitors (Sigma) and was sonicated followed by centrifugation at 14,000 g for 10 minutes. Proteins (50μg; Bradford assay) were separated on a NuPAGE Novex 4–12% Gel (Invitrogen) and transferred to a PVDF membrane (Millipore). Blots were incubated with polyclonal antibodies against EAAT2 (Millipore, ab1783, 1:2000), EAAT1 (Santa Cruz, sc-15316, 1:1000), GFAP(Sigma G3893, 1:25,000) and monoclonal mouse antibody against β-actin (Abcam, ab6276, 1:5000) followed by secondary IR-Dye 800CW donkey anti-guinea pig IgG antibody (Licor 1:10,000) or anti-mouse IgG antibody (Licor 1:20,000). The signals were visualized with a LI-COR Infrared Odyssey Scanner (LI-COR Biosciences, Lincoln, NE). Signal intensities were analyzed with Odyssey 2.1 software package by a rater (ML) blinded to the diagnosis. The signals of EAAT1 and EAAT2 were normalized to β-actin signals for each sample. Two sets of Western blots were repeated to obtain an average ratio of protein to β-actin value for each sample, expressed as relative to that of controls, arbitrarily set at 1.0. We also tested the specificity of EAAT1 and EAAT2 antibodies on Western blot using the peptide block method described above.

### Genotyping

We extracted DNA from frozen human brains using QIAamp DNA Mini kit (Qiagen) and we amplified the region containing rs3794087, an intronic variant of *SLC1A2*, by polymerase chain reactions (PCRs) using published primer sequences [4]. The PCR fragments were sent for commercial sequencing to determine the genotype of rs3794087.

### Data analyses

Data were analyzed in SPSS (v.21). All the continuous measures analyzed in this study were normally distributed. Demographic and clinical characteristics of ET cases and controls were compared using Student’s t –tests and Chi-square tests. For non-parametric comparisons, we used Mann-Whitney tests. Pearson’s correlation coefficients were used to assess correlations between continuous measures that were normally distributed. Spearman’s rank correlation coefficient was used to assess correlations between measures that were not normally distributed. We also used two-way ANOVA to analyze the EAAT2 levels in ET cases and controls with different rs3794087 genotypes.

## Results

### ET cases and controls

ET cases and controls had similar ages, gender, brain weights, PMI, and CERAD plaque scores. ET cases had higher PC axonal torpedo counts and a lower number of PCs (Table 1). All ET cases had tremor onset prior to age 65 years.

**Table 1:** **Clinical and pathological features of ET cases and control**

	**Western blot analysis**		**Immunohistochemistry**	
	**ET**	**Controls**	**ET**	**Controls**
**n**	**16**	**13**	**10**	**12**
Age at death (years)	83.6 ± 5.5	79.7 ± 7.1	85.7 ± 5.9	81.5 ± 6.4
Female gender	11 (68.8%)	8 (61.5%)	8 (80.0%)	10 (83.3%)
Age of tremor onset	30.9 ± 21.4	NA	42.0 ± 30.5	NA
Disease duration	53.4 ± 26.0	NA	44.3 ± 31.8	NA
Total tremor scores	23.8 ± 10.5	NA	24.8 ± 12.9	NA
Presence of head tremor	9 (56.3%)	NA	8 (80.0%)	NA
Presence of voice tremor	4 (25.0%)	NA	4 (40.0%)	NA
Family history of tremor	8 (50.0%)	NA	8 (80.0%)	NA
Brain weight (grams)	1170 ± 155	1181 ± 173	1138 ± 192	1174 ± 140
Postmortem interval (hours)	3.2 ± 4.7	5.9 ± 2.7	3.5 ± 5.8	9.7 ± 10.6
Axonal torpedoes	22.1 ± 20.7**	4.6 ± 7.7	12.7 ± 9.8**	6.8 ± 4.3
Purkinje Cell Count	6.5 ± 1.6**	10.2 ± 2.3	7.1 ± 1.6*	9.4 ± 2.2

*p < 0.05.

**p < 0.01.

### Specificity of EAAT1 and EAAT2 antibodies

We first assessed the specificity of EAAT1 antibody on Western blot and immunohistochemistry. EAAT1 antibody labeled a major 64 kDa band on Western blot, consistent with prior reports on EAAT1 using post-mortem human brain tissue [23,24]. The multimeric form of EAAT1 (190 kDa) and the differential glycosylation form of EAAT1 (97 kDa), which we observed, has also been noted by others [23,24]. A 55 kDa band of EAAT1 can represent the alternative spliced form of EAAT1 (Additional file 2: Figure S1A) [25]. Pre-incubation of EAAT1 antibody with EAAT1 peptide completely blocked all immunoreactive bands on Western blot. EAAT1 antibody also strongly labeled cerebellar cortex, and this immunoreactivity was entirely blocked by pre-incubation with EAAT1 peptide (Additional file 2: Figure S1B).

Next, we determined the specificity of EAAT2 antibody. EAAT2 antibody labeled a 55 kDa band on Western blot, consistent with prior studies (Additional file 3: Figure S2A) [23,24]. Several other studies have reported a slightly higher molecular weight (~60 kDa) for EAAT2, which might be due to differential splicing and/or glycosylation [24,26,27]. We also observed faint labeling of much higher molecular weight bands, suggesting that EAAT2 also forms multimers, as previously described (Additional file 3: Figure S2A) [23,24]. The EAAT2 antibody also labeled a similar 55 kDa band in a human cerebral cortical lysate (Additional file 3: Figure S2B). Pre-incubation of EAAT2 antibody with EAAT2 peptide robustly eliminated the EAAT2 band on Western blot (Additional file 3: Figure S2C) and EAAT2 immunoreactivity on immunohistochemistry (Additional file 3: Figure S2D).

To assess possible cross-reactivity between EAAT1 and EAAT2 antibodies, we performed EAAT1 peptide block with EAAT2 antibody and we found that EAAT1 peptide did not diminish EAAT2 immunohistochemical labeling (Additional file 3: Figure S2E). These results demonstrated the specificity of both EAAT1 and EAAT2 antibodies. Since the higher molecular weight multimers of EAAT1 and EAAT2 were not consistently detected, we analyzed the predominant strongly staining monomers of EAATs on Western blots.

### Decreased EAAT2 levels in ET cerebellum

**Figure 1:**
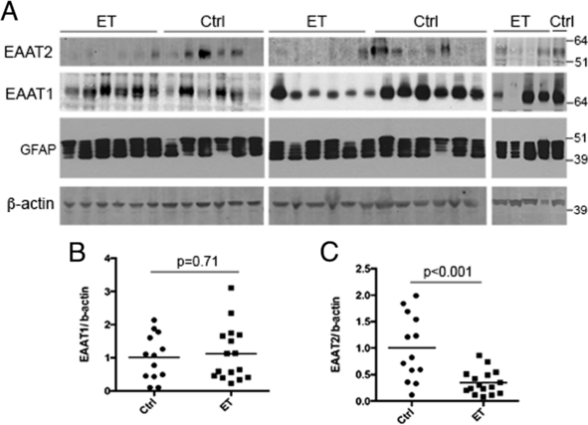
**Decreased EAAT2 protein levels in essential tremor (ET) cerebellar cortex.** Representative Western blots of EAAT1, EAAT2, GFAP, and β-actin **(A)** from cerebellar cortex of 16 ET cases and 13 controls. EAAT1 protein levels, normalized to a β-actin loading control, were similar between ET cases and controls **(B)**. EAAT2 protein levels were significantly decreased in ET cases compared to controls **(C)**. The fold-change in EAATs is expressed relative to control brains, which was arbitrarily set at 1.0. Mean ± SEM were shown.

We quantified EAAT1 and EAAT2 protein levels in the cerebellar cortex of ET cases and controls by Western blot. We found that EAAT1 levels in the cerebellar cortex were similar in ET cases and controls (1.12 ± 0.83 in ET cases vs. 1.01 ± 0.69 in controls, p = 0.71) (Figure 1A, B). By contrast, EAAT2 levels in ET cases were only 1/3 of that seen in controls (0.35 ± 0.23 in ET cases vs. 1.00 ± 0.62 in controls, p <0.001) (Figure 1A, C). To further investigate that the decrease in EAAT2 levels was not due to a general decrease in astrocytic proteins, we assessed the levels of another astrocytic protein: GFAP (Figure 1A). GFAP levels were similar in ET cases and controls (1.13 ± 0.33 in ET cases vs. 1.00 ± 0.22 in controls, p =0.26). We also normalized EAAT2 levels to GFAP levels in ET cases and controls, and we found that ET cases still had less than half the EAAT2 levels compared with controls (0.44 ± 0.52 in ET cases vs. 0.94 ± 0.80 in controls, p =0.02).

EAAT2 protein levels were positively correlated with PC counts (Pearson’s correlation coefficient r = 0.53, p = 0.004, i.e., individuals with lower EAAT2 levels had lower PC counts) (Additional file 4: Figure S3). EAAT2 levels tended to inversely correlate with torpedo counts; however, the association was only modest and did not reach statistical significance (r = -0.21, p =0.28). We further explored that whether EAAT2 levels were related to medication use. We found that EAAT2 levels did not differ between ET cases treated with and without propranolol (0.36 ± 0.23 vs. 0.42 ± 0.31, p =0.86), or treated with and without GABAergic medications (primidone or benzodiazepines) (0.32 ± 0.22 vs. 0.46 ± 0.29, p =0.36). None of the ET patients took glutamatergic medications such as topiramate. EAAT2 levels did not correlate with the number of ET medications (i.e. propranolol and primidone) (Spearman’s r = -0.33, p =0.11) or with the number of all medications (Pearon’s r = -0.02, p =0.95), suggesting that medication use alone could not account for the EAAT2 levels. EAAT2 levels did not correlate with the disease duration (Pearson’s r =0.19, p =0.50) or total tremor scores (r =0.32, p =0.27).

**Figure 2:**
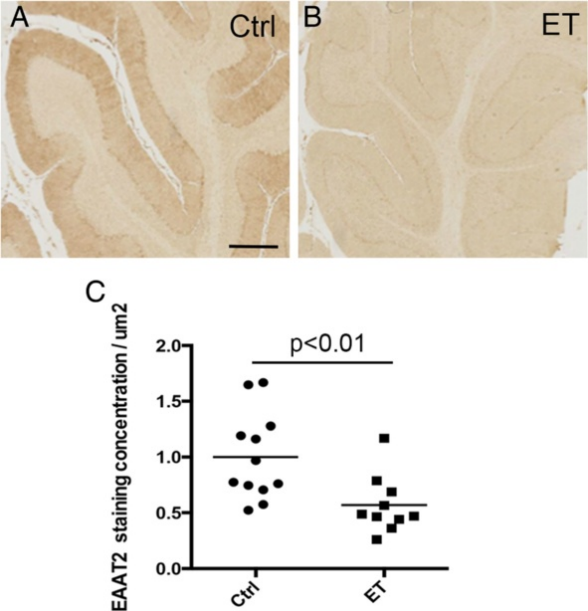
**Decreased intensity of EAAT2 immunohistochemistry in ET cerebellar cortex.** Paraffin-embeded sections immunolabeled with EAAT2 antibody were scanned and the intensity of immunostaining of the cerebellar cortex was quantified. The intensity was normalized to the randomly selected white matter regions. We found a significant decrease in EAAT2 immunoreactivity in the ET cerebellar cortex **(A-C)**. *Scale bar* 0.5 mm. Ctrl = control.

In the immunohistochemistry study, ET cases also had decreased EAAT2 levels when compared to controls (0.60 ± 0.25 in ET cases vs. 1.00 ± 0.41 in controls) (Figure 2A-C). The levels of EAAT2 on the Western blot correlated with those from EAAT2 immunohistochemistry (Pearson’s correlation coefficient r =0.62, p <0.01).

We also determined the genotypes of rs3794087 in ET cases and controls, although the number of subjects was too small to subject these analyses to statistical testing. We found that 50% of ET cases and 84.6% of controls had GG genotypes, 37.5% of ET cases and 15.4% of controls had GT genotypes, and 12.5% ET cases and no controls had TT genotypes (Additional file 5: Figure S4A). ET cases with both the GG and GT genotypes had significantly decreased EAAT2 levels than controls with the same genotypes (two way ANOVA, p <0.05 and p <0.01, respectively) (Additional file 5: Figure S4B).

### EAAT protein localization in cerebellar cortex

**Figure 3:**
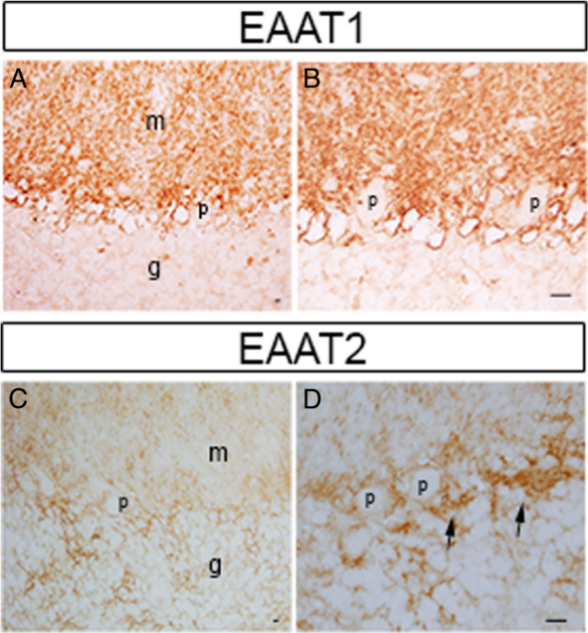
**EAAT expression in the cerebellar cortex.** Immunohistochemistry with anti-EAAT1 or anti-EAAT2 antibody in the cerebellar cortex. EAAT1 was expressed predominantly in the Bergmann glia radial processes in the molecular layer **(A, B)**. EAAT2 was expressed in the glia radial processes in the molecular layer and in the bushy astrocytes in the granular cell layer **(C, D)**. Purkinje cell and granule cell bodies did not express EAATs **(A-D)**. EAAT2 was also enriched in the astrocytic processes surrounding the PC axonal initial segment (**D**, arrows) m: molecular layer, g: granule cell layer, p: PC body. *Scale bar* 25 μm.

**Figure 4:**
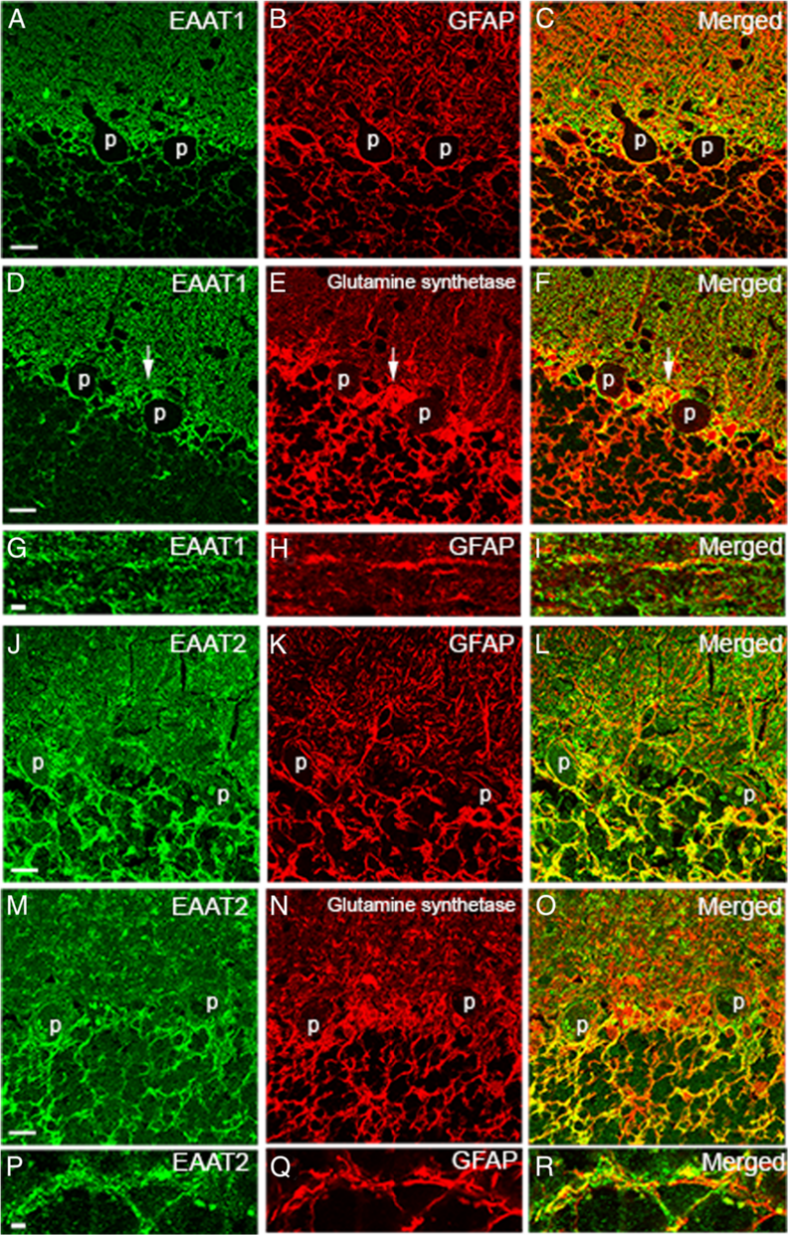
**EAATs colocalized with astrocytic markers.** Dual immunofluorescence labeling of ET cerebellar cortex with anti-EAAT1 or anti-EAAT2 (Alexa 488, green, **A**, **D**, **G**, **J**, **M**, **P**), anti-GFAP (Alexa 594, red, **B**, **E**, **K**, **Q**), and anti-glutamate synthetase (Alexa 594, red, **E**, **N**). Both EAAT1 and EAAT2 partially colocalized with GFAP or glutamine synthetase in the cerebellar cortex **(C, F, L, O)**. The cell bodies of Bergmann glia showed stronger colocalization with EAAT1 than with EAAT2 (arrows, in **D-F**). Higher magnification images showed both EAAT1 and EAAT2 form puncta along the GFAP-positive astrocytic processes **(G-I, P-R)**. p: PC body. *Scale bar* 25 μm.

We next investigated the cellular distribution of EAAT1 and EAAT2 proteins in the cerebellar cortex. We did not observe different expression patterns of EAAT1 and EAAT2 in ET cases and controls. Immunolabeling of EAATs in paraffin-embedded cerebellar cortical sections revealed that EAAT1 was expressed predominantly in the Bergmann glia radial processes in the molecular and far less so in the granule cell layer (Figures 3A, B; 4A, D). EAAT2 was distributed both in the glial processes in the molecular layer and also in the bushy astrocytes residing in the granule cell layer (Figures 3C, D; 4J, M), consistent with a previous report [28].

We performed dual immunoflourescence labeling of EAAT1 and EAAT2, and astrocytic markers, GFAP or glutamine synthetase, in the cerebellar cortex of ET cases and controls (Figure 4A-F, J-O). Both EAAT1 and EAAT2 appeared to be puncta along the GFAP- positive astrocytic processes (Figure 4G-I, P-R), showing that GFAP and glutamine synthetase were expressed predominantly in the cell bodies and proximal processes of astrocytes whereas EAATs were expressed in the end feet of the astrocytic processes [28]. There was stronger co-localization of EAAT1 and astroctyic markers in cytoplasm of Bergman glial cell soma than observed with EAAT2, consistent with greater expression of EAAT1 than EAAT2 in Bergmann glia, as previously described [29]. The EAAT2 expression in the granule cell layer extensively colocalized with glutamine synthetase and GFAP. These studies confirmed that EAAT1 and EAAT2 were expressed in astrocytic processes in the cerebellar cortex.

### EAAT2 and the PC axonal initial segment (AIS)

**Figure 5:**
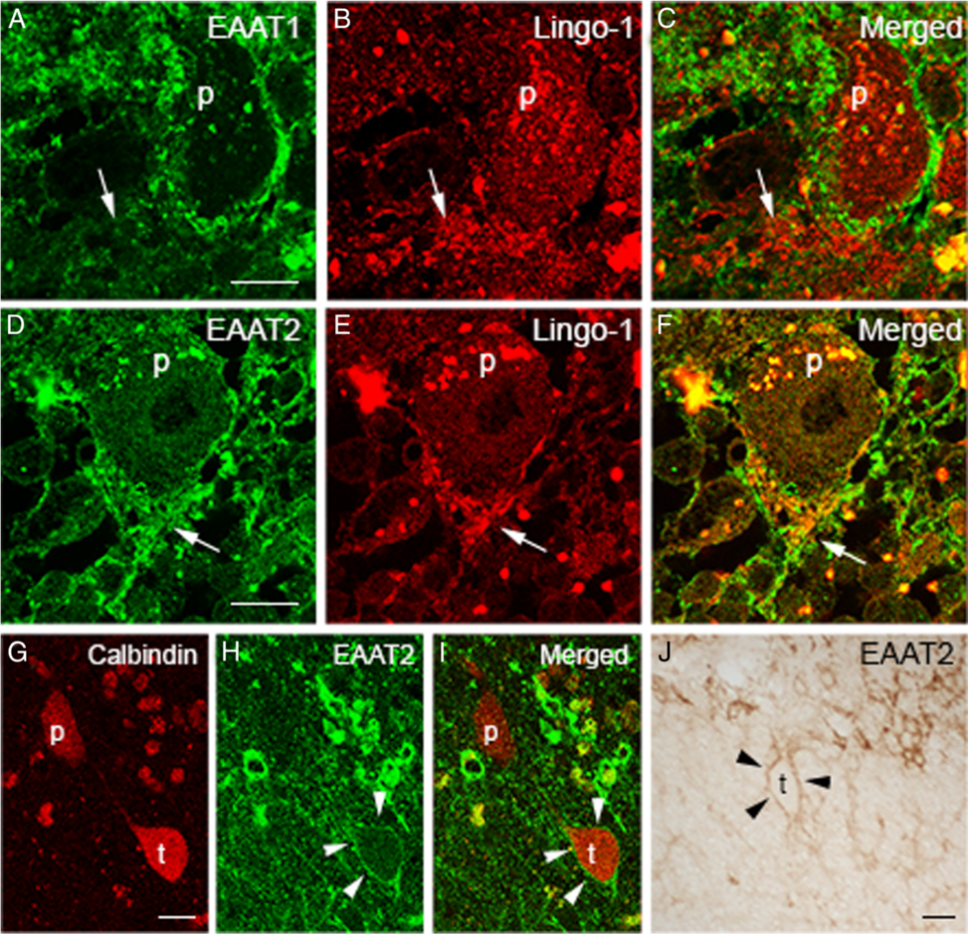
**EAATs in astrocytic processes surrounding PC axonal initial segment (AIS) in the ET cerebellar cortex.** Dual immunofluorescence with anti-EAAT1 or anti-EAAT2 (Alexa 488, green) antibody and anti-Lingo-1 (Alexa 594, red) antibody in the region adjacent to PC AIS **(A-F)**. Basket cell pinceau were visualized with anti-Lingo-1 antibody. We found that only very low levels of EAAT1 were found in this region (**A-C**, arrows). In contrast, EAAT2 positive astrocytic processes interdigitated with Lingo-1 positive basket cell processes, surrounding PC AIS (**D-F**, arrows). Dual immunofluorescence with anti-EAAT2 (Alexa 488, green) and anti-Calbindin (Alexa 594, red) antibody to investigate the relationship between PC axonal torpedoes and EAAT2-expressing astrocytic processes **(G-I)**. EAAT2-expressing astrocytic processes surrounded a PC axonal torpedo (Arrowheads, **H-I**). Immunohistochemistry with anti-EAAT2 antibody also revealed EAAT2-expressing astrocytic processes distributed around a PC axonal torpedo (Arrowheads, **J**). p: Purkinje cell body, t: PC axonal torpedo. *Scale bar* 25 μm.

In our postmortem studies of ET, we have identified several abnormal pathological features that are in proximity to the PC AIS, including torpedoes, “hairy” baskets, and elongated Lingo-1 labeled basket cell pinceaus [15,30,31]. The PC AIS is surrounded by astrocytic processes in addition to basket cell pinceau [32]. We investigated EAAT1 and EAAT2 expression in astrocytic processes in this region and around torpedoes identified the granule cell layer. We first labeled basket cell pinceau with Lingo-1 antibody and co-immunostained with EAAT1 or EAAT2 antibodies. We observed that EAAT2, but not EAAT1, labeled astrocytic processes interdigitated with Lingo-1 labeled basket cell processes (Figure 5A-F), suggesting that EAAT2 is the major glutamate transporter in this region. In addition, some bushy astroctyes in the granule cell layer showed stronger EAAT2 staining, and their processes were visualized surrounding PC axonal torpedoes and in close proximity to the PC axon distal to the torpedo (Figure 5G-J).

## Discussion

*SLC1A2* polymorphisms have been associated with ET [3-5], and *SLC1A2* encodes EAAT2, a major glutamate transporter in the adult brain [12]. In the present study of the postmortem cerebellar cortex, Western blot analysis demonstrated that EAAT2 levels were decreased in ET cases compared to controls, whereas EAAT1 levels were similar in ET cases and controls. EAAT1 and EAAT2 are expressed in astrocytes and interestingly, EAAT2, but not EAAT1, was present in the astrocytic processes surrounding the PC AIS, a site found to be particularly abnormal in prior studies of ET [15,30,31].

Functional astrocytes critically regulate glutamate levels at the synapses, and the membrane-bound glutamate transporters, EAAT1 and EAAT2, are important in this recycling of glutamate [8]. Failure of glutamate reuptake by astrocytes would lead to accumulation of glutamate in the synaptic cleft and over-stimulation of glutamate receptors. Over-excited neurons have elevated cytoplasmic calcium levels, which subsequently triggers neuronal injury or cell death [33]. This mechanism of excitotoxicity has been implicated in many neurological disorders, including epilepsy [9], stroke [10], amyotrophic lateral sclerosis [11], AD [34], traumatic brain injury [35], multiple sclerosis [36], and schizophrenia [23,24]. Interestingly, in ET, rhythmic over-excitation of glutamatergic olivo-cerebellar climbing fibers, which release glutamate onto PCs, has been hypothesized to be of possible patho-mechanistic importance, although this is purely hypothetical [37]. With the down-regulation of EAAT2 in the ET cerebellar cortex, the capacity of glutamate reuptake could be reduced.

Our current results should not be over-interpreted. They merely suggest that ET cerebella might be more vulnerable to excitotoxic damage than those of controls; they do not directly demonstrate greater vulnerability, nor do they show that there is excessive over-excitation derived from glutamatergic olivo-cerebellar climbing fibers in ET. EAAT2 levels correlated with PC counts but not tremor scores or tremor duration, providing further support that decreased EAAT2 levels in ET might cause excitotoxic PC death and might not be the result of long-standing tremor. Although climbing fiber - PC synapses in the molecular layer have been postulated as the location of PC excitotoxicity in ET [38], other glutamatergic synapses located more broadly throughout the cerebellar cortex, including parallel fiber-PC synapses, mossy fiber-granule neuron synapses, and climbing fiber collaterals to granule neurons and Golgi cells should be considered, and insufficient glutamate uptake in these synapses could result in over-excitation. In other words, increased vulnerability to excitotoxicity might not entirely be in the molecular layer in ET cases.

There are other possible explanations for our findings. One other explanation is that decreased EAAT2 levels also could be a secondary phenomenon due to the long-standing tremor in the olivo-cerebello-thalamic loop. Interestingly, harmine, a natural β-alkaloid that can induce ET-like tremor in animal models, can also alter the levels of EAAT2 in cultured human astrocytes [39], raising the possibility that the decreased EAAT2 levels could be the result of defective β-alkaloid homeostasis in ET [40].

Most of the discoveries in ET pathology focus on PCs [15,16]. In addition, structural changes in the region of the PC AIS, and adjacent areas, are observed in ET; these include torpedoes, “hairy” (i.e., hypertrophic) basket cell axonal processes, and elongated basket cell pinceau [15,30,31]. The PC AIS is surrounded by basket cell processes and astrocytic processes, and PC physiology is actively regulated by these structures. The PC AIS is critical for determining PC polarity and the timing of action potential firing [41], and structural alterations in this region thus might be directly related to ET. Two genetic polymorphisms have been linked to ET, *Lingo-1* and *SLC1A2* [3,42,43]*.* Interestingly, we found that Lingo-1 is enriched in the basket cell processes [31] whereas EAAT2 is selectively expressed in the astrocytic processes, both surrounding PC AIS. These two ET related proteins are enriched in this region, suggesting that the PC AIS and surrounding regions could be important in ET pathogenesis.

The limitation of the current study is that we focused only in the cerebellar cortex. First, future studies will need to elucidate whether EAAT2 levels are also altered in other brain regions such as the cerebellar dentate nucleus, thalamus and inferior olivary nucleus [44]. Also, there was available banked tissue for both Western blot and immunohistochemistry experiments in 8 ET case brains and 9 control brains; therefore, in some cases and controls tissue was available for only one type of experiment. Third, further studies to compare EAAT2 levels of ET cases with those of other tremor disorders such as tremor predominant Parkinson’s disease would be valuable. Finally, whether the down-regulation of EAAT2 can lead to increased PC vulnerability to excitotoxicity will need to be determined in future experiments.

## Conclusion

In summary, EAAT2 levels were significantly decreased in the ET cerebellar cortex, in contrast to similar levels of EAAT1 levels between ET cases and controls. ET brains might be more vulnerable to excitotoxic damage than those of controls. Further study of the relationship between astrocytes and PC injury might be central to understanding ET pathogenesis. Medications that increase EAAT2, such as β-lactam antibiotics [45], might be candidates for therapy for ET.

## Additional files

Additional file 1: Table S1. List of medications in essential tremor patients. Additional file 2: Figure S1. Specificity of EAAT1 antibody. EAAT1 antibody labeled a broad band of molecular weight around 64 kDa (A). EAAT1 multimers and the differential glycosylated form of a higher molecular weight could be occasionally seen (arrowheads, A). An alternative spliced form of EAAT1 could be seen at 55 kDa. Pre-incubation of EAAT1 antibody with EAAT1 peptide abolished immunoreactive bands on Western blot (A, left panel: EAAT1 antibody alone, right panel: EAAT1 antibody with EAAT1 peptide block). Immunohistochemistry of EAAT1 in the cerebellar cortex revealed immunoreactivity in the astrocytes mainly in the molecular layer, and EAAT1 peptide block eliminated the immunoreactivity in an adjacent paraffin-embedded section (B, upper panel: EAAT1 antibody alone, lower panel: EAAT1 antibody with EAAT1 peptide block). Additional file 3: Figure S2. Specificity of EAAT2 antibody. EAAT2 antibody labeled a strong band at the molecular weight of 55 kDa (arrowhead, A). Faint multimers of EAAT2 of higher molecular weight can also be observed (arrows, A). EAAT2 antibody also recognized the same band in the human cerebral cortex in ET cases (B). Preincubation of EAAT2 antibody with EAAT2 peptide abolished the 55 kDa EAAT2 band (C, left panel: EAAT2 antibody alone, right panel: EAAT2 antibody with EAAT2 peptide block). Immunohistochemistry of EAAT2 in the cerebellar cortex revealed immunoreactivity in the astrocytes in the molecular and granular cell layer and EAAT2 peptide diminished the immunoreactivity in an adjacent paraffin-embedded section (D, left panel: EAAT2 antibody alone, right panel: EAAT2 antibody with EAAT2 peptide block). Preincubation with EAAT1 peptide with EAAT2 antibody did not eliminate the EAAT2 immunoreactivity in an adjacent paraffin-embedded section (E, left panel: EAAT2 antibody alone, right panel: EAAT2 antibody with EAAT1 peptide block). Additional file 4: Figure S3. EAAT2 levels and PC counts. EAAT2 levels and PC counts of 16 ET cases and 13 controls were shown. Dots of different colors represented different PC count tertiles. The bars represented the mean of the EAAT2 levels in each tertile group. Additional file 5: Figure S4. EAAT2 levels and rs3794087 genotypes. We determined genotypes using frozen brain tissue of 16 ET cases and 13 controls, and the percentage of ET cases and controls with each genotype is shown (A). Also, the number in each group is shown inside each bar (A). ET cases had significantly decreased EAAT2 levels as compared to controls in both GG and GT genotype groups (B). There were no controls in the TT group, so no comparison was possible. Mean ± SEM were shown.
