# Supplementary material for: Decreased EAAT2 protein expression in the essential tremor cerebellar cortex
Source: Acta Neuropathol Commun. 2014 Nov 13;2:157. doi: 10.1186/s40478-014-0157-z (PMC4239402; doi:10.1186/s40478-014-0157-z)
Supplement: Additional file 1: Table S1. — List of medications in essential tremor patients. [file 40478_2014_157_MOESM1_ESM.doc]

**Supplemental Table 1. List of medications in essential tremor patients**

| Subject | WB | IHC | Beta blocker | Primidone | Topiramate | Other medication |
| --- | --- | --- | --- | --- | --- | --- |
| 1 | + |  | - | - | - | - |
| 2 | + | + | + | - | - | levothyroxin, amiodarone |
| 3 | + | + | + | - | - | - |
| 4 | + |  | + | - | - | simvatatin, diltiazem, ursodeoxycholic acid, prazosin, hydroxizine |
| 5 | + |  | - | + | - | atorvastatin, paroxetine, travapost, alendronate |
| 6 | + |  | + | + | - | allopurinol, warfarin, aspirin, furosamide, hydrocodone, isosorbide mononitrate, levothyroxine, nortriptyline, pravastatin |
| 7 | + |  | + | + | - | rofecoxib, amitriptyline, escitalopram, atorvastatin, alendronate |
| 8 | + |  | + | - | - | escitalipram, clopidogrel, travaprost, pirbuterol, alendronate, oxybutynin |
| 9 | + | + | + | + | - | furosemide |
| 10 | + | + | + | - | - | donepezil, memantine, citalopram, ramipril, pravastatin, fexofenadine, quinine |
| 11 | + | + | + | + | - | raloxifene, lovastatin, levothyroxine |
| 12 | + | + | - | + | - | doxazosin, levothyroxine nitroglycerin, trazodone, furosamide, amlodipine |
| 13 | + | + | + | + | - | propoxyphene, sertraline, lisinopril, furosamide, alendronate, cyanocopalamin |
| 14 | + | + | - | - | - | alprazolam, simvastatin, omeprazole, escitalopram |
| 15 | + |  | + | - | - | hydrochlorothiazide |
| 16 | + |  | + | + | - | omeprazole, amiodarone, citalopram, digoxin, trazodone |
| 17 |  | + | + | + | - | rofecoxib, amitriptyline, escitalopram, atorvastatin, alendronate |
| 18 |  | + | - | - | - | - |

WB: cases used for Western blot analysis

IHC: cases used for immunohistochemistry analysis
