# Supplementary figures and images for: Decreased EAAT2 protein expression in the essential tremor cerebellar cortex
Source: Acta Neuropathol Commun. 2014 Nov 13;2:157. doi: 10.1186/s40478-014-0157-z (PMC4239402; doi:10.1186/s40478-014-0157-z)

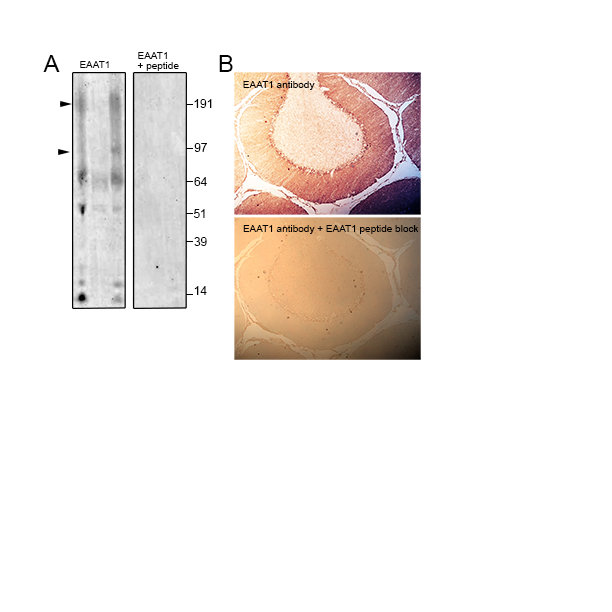

Supplement: Additional file 2: Figure S1. — Specificity of EAAT1 antibody. EAAT1 antibody labeled a broad band of molecular weight around 64 kDa (A). EAAT1 multimers and the differential glycosylated form of a higher molecular weight could be occasionally seen (arrowheads, A). An alternative spliced form of EAAT1 could be seen at 55 kDa. Pre-incubation of EAAT1 antibody with EAAT1 peptide abolished immunoreactive bands on Western blot (A, left panel: EAAT1 antibody alone, right panel: EAAT1 antibody with EAAT1 peptide block). Immunohistochemistry of EAAT1 in the cerebellar cortex revealed immunoreactivity in the astrocytes mainly in the molecular layer, and EAAT1 peptide block eliminated the immunoreactivity in an adjacent paraffin-embedded section (B, upper panel: EAAT1 antibody alone, lower panel: EAAT1 antibody with EAAT1 peptide block). [file 40478_2014_157_MOESM2_ESM.tiff]

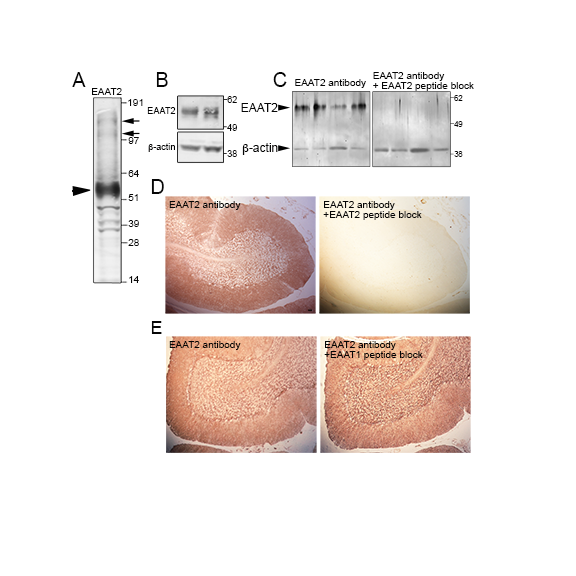

Supplement: Additional file 3: Figure S2. — Specificity of EAAT2 antibody. EAAT2 antibody labeled a strong band at the molecular weight of 55 kDa (arrowhead, A). Faint multimers of EAAT2 of higher molecular weight can also be observed (arrows, A). EAAT2 antibody also recognized the same band in the human cerebral cortex in ET cases (B). Preincubation of EAAT2 antibody with EAAT2 peptide abolished the 55 kDa EAAT2 band (C, left panel: EAAT2 antibody alone, right panel: EAAT2 antibody with EAAT2 peptide block). Immunohistochemistry of EAAT2 in the cerebellar cortex revealed immunoreactivity in the astrocytes in the molecular and granular cell layer and EAAT2 peptide diminished the immunoreactivity in an adjacent paraffin-embedded section (D, left panel: EAAT2 antibody alone, right panel: EAAT2 antibody with EAAT2 peptide block). Preincubation with EAAT1 peptide with EAAT2 antibody did not eliminate the EAAT2 immunoreactivity in an adjacent paraffin-embedded section (E, left panel: EAAT2 antibody alone, right panel: EAAT2 antibody with EAAT1 peptide block). [file 40478_2014_157_MOESM3_ESM.tiff]

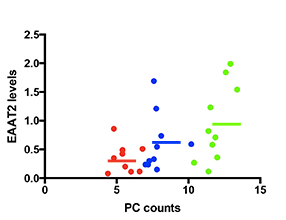

Supplement: Additional file 4: Figure S3. — EAAT2 levels and PC counts. EAAT2 levels and PC counts of 16 ET cases and 13 controls were shown. Dots of different colors represented different PC count tertiles. The bars represented the mean of the EAAT2 levels in each tertile group. [file 40478_2014_157_MOESM4_ESM.tiff]

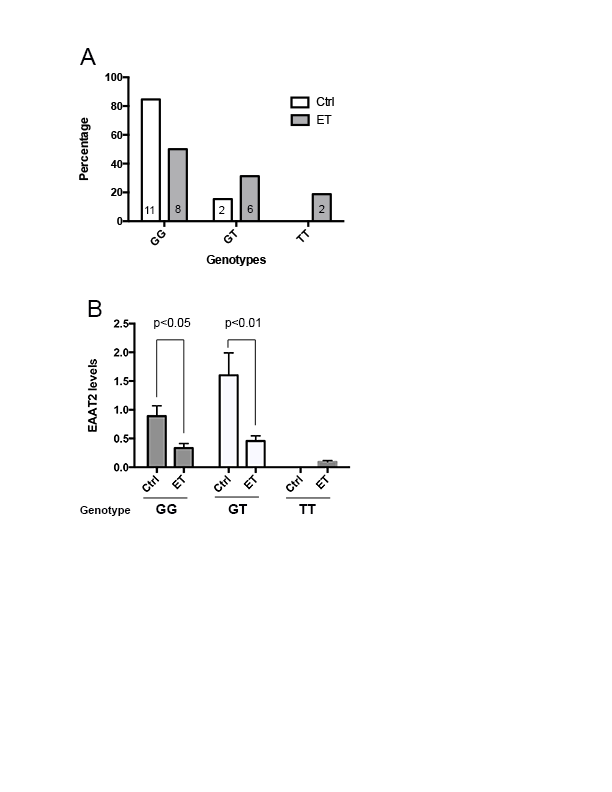

Supplement: Additional file 5: Figure S4. — EAAT2 levels and rs3794087 genotypes. We determined genotypes using frozen brain tissue of 16 ET cases and 13 controls, and the percentage of ET cases and controls with each genotype is shown (A). Also, the number in each group is shown inside each bar (A). ET cases had significantly decreased EAAT2 levels as compared to controls in both GG and GT genotype groups (B). There were no controls in the TT group, so no comparison was possible. Mean ± SEM were shown. [file 40478_2014_157_MOESM5_ESM.tiff]
